# Supplementary material for: The Systems Biology Research Tool: evolvable open-source software
Source: BMC Syst Biol. 2008 Jun 29;2:55. doi: 10.1186/1752-0509-2-55 (PMC2446383; doi:10.1186/1752-0509-2-55)
Supplement: Additional file 1 — SBRT Archive. An archive of the current version of the Systems Biology Research Tool. [file 1752-0509-2-55-S1.zip › sbrt-1.4.0/doc/developers_guide/api/sbrt/kernel/fba/proc/CdHarFluxVectorProcess.html]

CdHarFluxVectorProcess


|  |  |  |
| --- | --- | --- |
| **Overview** \| **Package** \| **Class** \| **Use** \| **Tree** \| **Deprecated** \| **Index** \| **Help** | |  |
| **PREV CLASS**   **NEXT CLASS** | **FRAMES**    **NO FRAMES**     **All Classes** |  |
| SUMMARY: NESTED \| FIELD \| CONSTR \| METHOD | DETAIL: FIELD \| CONSTR \| METHOD |  |


---


## sbrt.kernel.fba.proc Class CdHarFluxVectorProcess

```
java.lang.Object
  sbrt.kernel.fba.proc.FbaProcess<CdHarFluxVectorComputer.Results>
      sbrt.kernel.fba.proc.CdHarFluxVectorProcess
```

**All Implemented Interfaces:**: KernelProcess<CdHarFluxVectorComputer.Results>, MonitoredProcess<CdHarFluxVectorComputer.Results>

---

``` public final class CdHarFluxVectorProcess extends FbaProcess<CdHarFluxVectorComputer.Results> implements MonitoredProcess<CdHarFluxVectorComputer.Results> ```

This class is used to perform coordinate direction hit-and-run flux vector
experiments.

**Author:**
:   This class was written and documented by Jeremiah Wright while in
    the Wagner lab.

---

**Constructor Summary**

`CdHarFluxVectorProcess(CdHarFluxVectorComputer computer)`
  
          Constructs a new coordinate direction hit-and-run flux vector
experiment.


| **Method Summary** | |
| --- | --- |
| `Fluxome` | `getFluxome()`             Returns the fluxome for which flux vectors are to be generated. |
| `int` | `getIterations()`             Returns the total number of flux vectors to be generated. |
| `int` | `getMaxWastedMoves()`             Returns the allowed number of wasted moves. |
| `int` | `getPointsPerPoint()`             Returns the number of points generated per points returned. |
| `ProgressMonitor` | `getProgress()`             Returns a progress monitor for this experiment. |
| `void` | `run(ResultsManager<CdHarFluxVectorComputer.Results> resultsManager)`             Generates the specified number of random flux vectors and adds each to the provided results manager. |
| `void` | `setIterations(int iterations)`             Sets the total number of flux vectors to be generated. |
| `void` | `setMaxWastedMoves(int maxWastedMoves)`             Sets the maximum number of wasted moves to allow. |
| `void` | `setPointsPerPoint(int pointsPerPoint)`             Sets the number of points to generate for each point added to the specified results manager. |

**Methods inherited from class sbrt.kernel.fba.proc.FbaProcess**

`toString`

**Methods inherited from class java.lang.Object**

`clone, equals, finalize, getClass, hashCode, notify, notifyAll, wait, wait, wait`

**Constructor Detail**

### CdHarFluxVectorProcess

```
public CdHarFluxVectorProcess(CdHarFluxVectorComputer computer)
```

:   Constructs a new coordinate direction hit-and-run flux vector
    experiment.

    **Parameters:**: `computer` - the computer with which all flux vectors will be generated.


**Method Detail**

### setIterations

```
public void setIterations(int iterations)
```

:   Sets the total number of flux vectors to be generated.

    :   **Parameters:**: `iterations` - the total number of flux vector to be generated.

---


### setMaxWastedMoves

```
public void setMaxWastedMoves(int maxWastedMoves)
```

:   Sets the maximum number of wasted moves to allow.

    :   **Parameters:**: `maxWastedMoves` - the number of wasted moves to allow.

---


### setPointsPerPoint

```
public void setPointsPerPoint(int pointsPerPoint)
```

:   Sets the number of points to generate for each point added to the
    specified results manager.

    :   **Parameters:**: `pointsPerPoint` - the number of points to generate for each point added to the specified results manager.

---


### getIterations

```
public int getIterations()
```

:   Returns the total number of flux vectors to be generated.

    :   **Returns:**: the total number of flux vectors to be generated.

---


### getMaxWastedMoves

```
public int getMaxWastedMoves()
```

:   Returns the allowed number of wasted moves.

    :   **Returns:**: the allowed number of wasted moves.

---


### getPointsPerPoint

```
public int getPointsPerPoint()
```

:   Returns the number of points generated per points returned.

    :   **Returns:**: the number of points generated per points returned.

---


### getFluxome

```
public Fluxome getFluxome()
```

:   Returns the fluxome for which flux vectors are to be generated.

    :   **Specified by:**: `getFluxome` in class `FbaProcess<CdHarFluxVectorComputer.Results>`
    :   **Returns:**: the fluxome for which flux vectors are to be generated.

---


### getProgress

```
public ProgressMonitor getProgress()
```

:   Returns a progress monitor for this experiment.

    :   **Specified by:**: `getProgress` in interface `MonitoredProcess<CdHarFluxVectorComputer.Results>`
    :   **Returns:**: a progress monitor for this experiment.

---


### run

```
public void run(ResultsManager<CdHarFluxVectorComputer.Results> resultsManager)
```

:   Generates the specified number of random flux vectors and adds each to
    the provided results manager.

    :   **Specified by:**: `run` in interface `KernelProcess<CdHarFluxVectorComputer.Results>`
    :   **Parameters:**: `resultsManager` - the results manager to which all computed vectors will be added.


---


|  |  |  |
| --- | --- | --- |
| **Overview** \| **Package** \| **Class** \| **Use** \| **Tree** \| **Deprecated** \| **Index** \| **Help** | |  |
| **PREV CLASS**   **NEXT CLASS** | **FRAMES**    **NO FRAMES**     **All Classes** |  |
| SUMMARY: NESTED \| FIELD \| CONSTR \| METHOD | DETAIL: FIELD \| CONSTR \| METHOD |  |


---
